# “Our fear is finished,” but nothing changes: efforts of marginalized women to foment state accountability for maternal health care in a context of low state capacity

**DOI:** 10.1186/s12889-019-7028-2

**Published:** 2019-06-11

**Authors:** Marta Schaaf, Jashodhara Dasgupta

**Affiliations:** 1 Program on Global Health Justice and Governance, Heilbrunn Department of Population and Family Health (HDPFH), Mailman School of Public Health, Columbia University, 60 Haven Ave, B3, New York, NY 10032 USA; 2 National Foundation for India, Core 4A (Upper Ground Floor), India Habitat Centre, Lodi Road, New Delhi, 110003 India

**Keywords:** Social accountability, Collective action, Corruption, Global health, India, Gender

## Abstract

**Background:**

Women in India are often asked to make informal payments for maternal health care services that the government has mandated to be free. This paper is a descriptive case study of a social accountability project undertaken by SAHAYOG, a nongovernmental organization in Uttar Pradesh, India. SAHAYOG worked with community-based organizations and a grassroots forum comprised of low caste, Muslim, and tribal women to decrease the prevalence of health provider demands that women and their families make informal payments.

**Methods:**

The study entailed document review; interviews and focus group discussions with program implementers, governmental stakeholders, and community activists; and participant observation in health facilities.

**Results:**

The study found that SAHAYOG adapted their strategy over time to engender greater empowerment and satisfaction among program participants, as well as greater impact on the health system. Participants gained knowledge resources and agency; they learned about their entitlements, had access to mechanisms for complaints, and, despite risk of retaliation, many felt capable of demanding their rights in a variety of fora. However, only program participants seemed successfully able to avoid making informal payments to the health sector; health providers still demanded that other women make payments. Several features of the micro and macro context shaped the trajectory of SAHAYOG’s efforts, including deeply rooted caste dynamics, low provider commitment to ending informal payments, the embeddedness of informal payments, human resources scarcity, and the overlapping private interests of pharmaceutical companies and providers.

**Conclusion:**

Though changes were manifest in certain fora, providers have not necessarily embraced the notion of low caste, tribal, or Muslim women as citizens with entitlements, especially in the context of free government services for childbirth. Grassroots advocates, CBOs, and SAHAYOG assumed a supremely difficult task. Project strategy changes may have made the task somewhat less difficult, but given the population making the rights claims and the rights they were claiming, widespread changes in demands for informal payments may require a much larger and stronger coalition.

## Background

Gender and other social hierarchies shape vulnerability to ill health, as well as the quality of health care one receives [1]. Building the community consciousness, confidence, coalitions, and know-how for low status women to effectively claim maternal health and citizenship rights is the work of long-term, broad-based social movements. We examine the contribution of one campaign to this movement in Uttar Pradesh, India. Our study adds to the growing literature on social accountability and health by examining a long-term campaign tackling the particularly intransigent issue of informal payments for health care.

Despite high-level rhetorical commitment to improving maternal and child health in India, in many contexts, the quality of care in the public system can be quite poor [2–5]. Central and state level governments have put enormous effort into encouraging and incentivizing women to give birth in government health facilities, under the assumption that higher rates of institutional delivery would lead to decreased maternal and neonatal mortality rates [6]. However, overwhelmed health facilities are not necessarily able to provide quality healthcare to all women seeking it. Despite increased rates of institutional delivery, the maternal mortality rate has not changed significantly [7–9]. In the extreme, disproportionate emphasis on institutional delivery can yield “safe, yet violent” deliveries, where women experience disrespect and abuse during labor and delivery, and health care workers strain to fulfill their professional mandate in facilities with poor infrastructure, frequent stock outs, and punitive management [8, 10, 11].

In this context, health care providers often ask women to make informal payments for maternal health care services that the central or state government has mandated to be free. Informal payments are defined as “a direct contribution, which is made in addition to any contribution determined by the terms of entitlement, in cash or in-kind, by patients or others acting on their behalf, to health care providers for services to which patients are entitled” [12]. Deeply embedded in the way health systems function and resistant to quick fixes, informal payments can be financially significant for poor women and their families, and they may be demanded in a coercive manner that undercuts satisfaction, future utilization of the health system, and health citizenship [8, 13–16].

Policy-makers, activists, and donors state that social accountability efforts can help to end informal payments and disrupt the structures that give rise to them [17, 18]. Social accountability entails collective action among civil society actors to hold the state to account for failures to provide public goods, such as health care [19].

This paper is a descriptive, contextualized case study of a social accountability project undertaken by SAHAYOG, a non-governmental organization (NGO), partner CBOs, and a grassroots women’s form in Uttar Pradesh, India. The project, called My Health, My Voice (*Mera Swasthya, Meri Aawaz* in Hindi, or MS, MA) ran from January 2012 to June 2016, and sought to decrease demands by health providers that women and their families make informal payments for maternal health care.

### Social accountability

Social accountability builds on the longstanding field of participation in development, including in primary health care, by linking participation to the accountability principles of answerability and sanctions [20–22].

Several empirical articles and two reviews address the impact that social accountability efforts can have on maternal health and health more broadly. Researchers have found that in some contexts, social accountability campaigns can enhance knowledge and empowerment among community members; improve the clinical and interpersonal quality of service delivery; enhance trust between communities and the health system; further health system compliance with national guidelines regarding the availability of important inputs; and enhance the functioning of government- and community-supported institutions, such as Village Health Committees [17, 23–27]. Reduced demands for informal payments are among the results documented, but there has been very little research focused on social accountability and informal payments.

The degree to which social accountability activities affect citizen empowerment and health system governance depends in part on intent; some efforts simply seek to teach citizens to be more educated consumers of services, whereas others seek to “deepen democracy” by changing the terms of interactions between citizens and the state [28, 29]. Deepening democracy requires supporting inclusive, deliberative processes and the development of leaders from marginalized groups [30].

Jonathan Fox and Anuradha Joshi, two influential accountability researchers, make important distinctions within social accountability efforts addressing health and other service delivery areas. Fox describes the differences between strategic and tactical approaches, and Joshi and Houtzager, between widgets and watchdogs. Tactical approaches are bounded and are limited to “society-side” efforts to gather and project citizen voice. In contrast, strategic approaches entail multiple tactics, foster an enabling environment for collective action, and coordinate with synergistic efforts to improve state capacity to respond to citizen voice [31].

Joshi and Houtzager [19] describe widgets as “labelled mechanisms” which have been introduced by external actors. They differentiate these labelled mechanisms from watchdogs; civil society functions as a watchdog when there is ongoing “political engagement by social actors with the state as a part of a long-term pattern of interaction shaped both by historical forces and the current context” (p. 146).

Fox and Joshi and Houtzager conclude that the transformative – deepening democracy - potential of social accountability efforts is much greater when strategic, watchdog approaches are adopted, a conclusion that has been supported by other empirical work [32].

### Study context

India has a recent record of state-mandated, institutionalized processes to foster citizen participation; some of these processes are specific to the health sector. For example, the National Rural Health Mission (NRHM, now known as National Health Mission or NHM), a national effort to improve health care in rural areas, included community monitoring of service quality. This large-scale effort contributed to increased health provider and administrator acceptance of community monitoring and engagement [33]. However, Uttar Pradesh did not initially implement this aspect of the NRHM.

As compared to the rest of India, the state of Uttar Pradesh stands out for especially entrenched patriarchal, religious, and caste hierarchies [14, 34, 35]. Poor women have limited decision-making power, access to education, and freedom of movement [35, 36]. Many social and economic indicators are significantly worse in Uttar Pradesh than the median levels in India; maternal mortality is no exception, with an overall rate of 258 maternal deaths per 100,000 live births, as compared to the overall rate of 178 per 100,000 live births [8, 37]. These indicators also differ significantly across axes of inequity, with low caste, Muslim religion, lower education levels, and rural residence associated with worse maternal health outcomes and quality of care [3–5]. Caste divisions are sometimes at the core of political discourse and public sector operations. Political reputations are often based not on the delivery of services, but on the delivery of the fruits of patronage politics, such as employment, to one’s fellow caste members and political allies [36, 38, 39]. These factors make participatory processes based on the premise of universal, quality service delivery quite challenging. NGO efforts to deepen democracy by supporting leadership among the marginalized, fostering civil society watchdogs, and implementing strategic social accountability efforts face an especially challenging context.

SAHAYOG began working in Uttar Pradesh in 1992. An intermediate level organization, SAHAYOG ‘brings the state to the grassroots’ by educating women about their entitlements and political processes, and they bring the ‘grassroots to the state’ by facilitating women’s input into monitoring, agenda-setting, and policy-making forums.

MSAM (Mahila Swasthya Adhikar Manch or the Women’s Health Rights Forum) has been a fulcrum of SAHAYOG’s work since 2006. MSAM is comprised of approximately 12,000 largely illiterate, Muslim, scheduled caste, or tribal women from eight districts of Uttar Pradesh. MSAM members monitor public services and entitlements related to priority issues they themselves have identified, collate their findings into reports, and present these reports at district and state level dialogue events. In 2012, MSAM identified informal payments for maternal health care as a priority issue. SAHAYOG, CBOs, and MSAM members then conducted a survey in 11 districts, where women who had given birth in the past 6 months reported paying an average of 1277 rupees for maternal health services that were mandated to be free. One thousand two hundred and seventy seven rupees is the equivalent of $24 at the time the data were collected; in 2011–2012, the average wage for a daily laborer in rural UP was 135 rupees [40].

In response, SAHAYOG and the CBOs launched MS, MA, building on longstanding collaborations among SAHAYOG, the CBOs, and MSAM. The MS, MA project was enabled in part by the Government of Uttar Pradesh asking SAHAYOG and other civil society groups to monitor the implementation of Janani Shishu Suraksha Karyakaram (JSSK), a scheme ensuring free comprehensive maternity care. In partnership with the CBOs, SAHAYOG provided MSAM with training and facilitation support for regular member meetings, facilitated opportunities for them to meet with policy-makers in Lucknow, and supported the CBOs to reflect periodically on their work and adapt their strategies of engagement as appropriate.

It was a 4-year project wherein women used interactive voice response (IVR) on mobile phones to call a hotline and report having been asked to make an informal payment for maternal health care. The complaints were categorized by type and amount and were mapped and displayed on a website in real time. The project was started in 2 pilot districts and eventually scaled up to 7 districts.

**Fig. 1:**
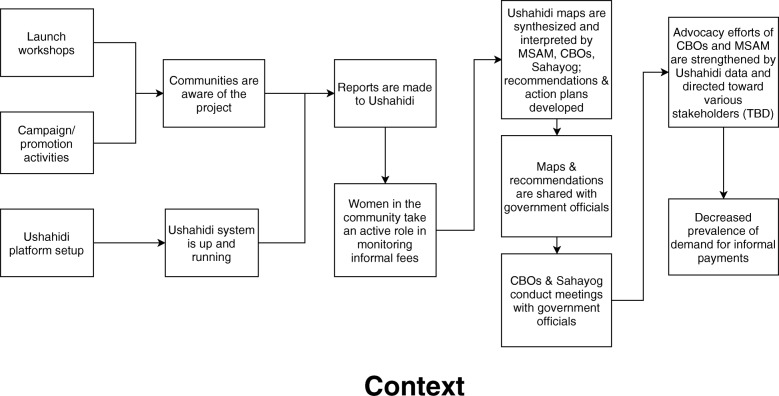
Initial MS, MA Theory of Change

With facilitation from SAHAYOG, members of MSAM and the CBOs articulated the theory of change depicted in Fig. 1 at the start of the project.

SAHAYOG, the CBOs, and MSAM believe that informal payments are a systems problem requiring systems change. Thus, the intent of the project was to decrease the prevalence of informal payments by demonstrating the scope of the phenomenon, as well as patterns associated with particular geographic areas and facilities, rather than to punish any particular provider or obtain remedy for a particular patient.

## Methods

The research question we sought to address in our case study was: What strategies did SAHAYOG employ to support CBOs and MSAM to reduce informal payments, and how did these strategies affect informal payments in a context of low state capacity and commitment to respond?

We undertook a descriptive case study, aiming to fully describe program strategies and to examine the outcomes in the context of larger “how” questions. This descriptive approach is justified partly by the fact that much of the extant research on social accountability in the health sector has focused more narrowly on externally-induced, discrete, time-bound interventions and outcomes without attendant exploration of program context and evolving program strategy. As a result, the peer-reviewed literature on social accountability for health does not adequately reflect the rich experiences of many national and sub-national NGOs. Yet, long-term, grassroots focused efforts that take an adaptive, context-specific programming approach may be germane to the strategic, watchdog approaches that contribute to transformational change. Both authors of the present study were part of a larger team that used Most Significant Change methodology to study the impact of the first two years of this project [14]. Through this evaluation, we identified specific contextual and organizational factors for further investigation. These factors included state willingness and capacity to respond to citizen demands; SAYAHOG’s evolving reputation and relationship with the state; MSAM relationships with health facilities; and health provider perceptions of informal payments [14, 41].

We collected data from two project districts: (1) Azamgarh, with an approximate population of 2.3 million women, and, (2) Mirzapur, with an approximate population of 1.2 million women [42]. The criteria for choosing these districts were: MSAM active in the site, high rates of reporting over time, anecdotal reports of decreased frequency in informal payments, and functioning CBO relationship with the government health authorities. The CBO partner’s relationship to the government health authorities in the districts is important for facilitating entry into the field, and these relationships were considered to be ‘very good’ in Azamgarh and ‘average’ in Mirzapur. Thus, we were confident that planned research activities in health facilities could take place. Mirzapur and Azamgarh differ in that Mirzapur is more geographically remote and has poorer socio-economic and gender equity indicators [37].

Within the districts of Azamgarh and Mirzapur, we intended our sampling strategy to result in the selection of typical (“common,” in case study terms), cases where there had been successes. We chose sites that would be representative of those places where the project was taken up by the population (women made reports), and where at least some minimal governmental buy-in was in evidence (anecdotal reports of response to the project, CBO has a functional relationship with the government health authorities, and the Medical Officers in Charge of the relevant facilities were willing to participate). We started the research when the project had already been implemented for several years. At that point, MSAM, the CBOs, and SAHAYOG had already concluded that this project was less successful than other efforts. Therefore, they wanted to focus research on understanding the successes they had, so that they could apply this knowledge to future programming.

Analysis of strategic, watchdog approaches requires research that accommodates complexity and that is attentive to explicit and implicit power by addressing the macro and the micro context that comprise the ‘accountability ecosystem’ [30]. We accomplished this by exploring a wide range of questions via several different qualitative tools and several days of observation. More specifically, we undertook:

- In depth, structured Key Informant Interviews (KII) of program implementers (*n* = 4).
- In-depth, structured, interviews (IDI) with governmental stakeholders at health facility and district levels (*n* = 7).
- Focus group discussions (FGDs) about experiences as a professional/community activist with MSAM members and with CBO staff (2 FGDs with the CBOs, and 6 FGDs with MSAM women, with a total of 68 people).
- Participant observation in healthcare facilities. Participant observation was carried out for 3 days in a primary health center in Mirzapur, 1 day in a district hospital in Mirzapur, 4 days in a primary health center in Azamgarh, and 1 day in a district hospital in Azamgarh. All of these facilities provide prenatal care and labor and delivery services. The actual observation entailed observing public areas of hospitals and having informal conversations with staff, providers, and patients to understand the norms guiding formal and informal financial transactions for maternal health care. Participant observation is particularly suited to this context as it allows for exploration of the “socially acquired and shared knowledge” of different stakeholders, thus facilitating understanding of the day-to-day realities [43]. Participant observation also provides some corrective to the reporting bias inherent in interview methods; it “can help to overcome the discrepancy between what people say and what they actually do” [43–45].
- Document review**.** Documents included government orders related to informal payments in the past 5 years from district/state health offices, official government comments/press releases available in the public domain, project documents and meeting summaries from SAHAYOG, and data from the My Health, My Voice website.

KII, IDI, and FGD guides were developed based on the social accountability, participation, and informal payment literature (deductive) as well as the research questions.. The questions probed interviewee experiences with informal payments, their interactions with others (e.g. health providers, facility managers) regarding payments, their understanding of the context and drivers of informal payments, and their engagement with MS, MA, among other areas. Representatives of SAHAYOG commented on the tools. All tools were pre-tested and revised as needed. With the exception of the FGDs with MSAM members and CBO staff, all study activities were undertaken by an Indian physician and Columbia University doctoral student in socio-medical sciences. JD, the Founding Director of SAHAYOG, conducted the CBO FGDs. As she is acknowledged as a trusted ally among the CBO participants, her facilitation of the CBO focus groups created an open atmosphere that helped to yield rich discussion.

Investigators approached potential participants, explained the purpose of the study according to a prepared recruitment script, and explained and provided information sheets in the local language. The Institutional Review Board of the Columbia University Medical Center and Sigma IRB in Delhi gave ethical clearance for the study including verbal consent.

KII, IDI, and FGD transcripts were transcribed, translated, and saved in Nvivo 11. A native Hindi speaker checked the quality of the translations. Detailed notes were written in English during the course of participant observation; these were written during quiet periods in the health facility and at the end of the day. These notes were also saved in Nvivo 11.

The transcripts and notes were analyzed using thematic coding. The initial list of analytic thematic codes was based on relevant theoretical and empirical literature related to social accountability, participation, and informal payments. Codes were modified following close review of all transcripts and discussion with JD, such that the final list included inductively and deductively derived codes [46, 47]. MS coded the data. Once the data were coded, MS developed thematic memos exploring the range and number of exemplars for the same codes, illustrating dimensions of the phenomena of interest [48, 49]. These memos included comparisons within and across interlocutors, noting approximately how many types of each interlocutor shared opinions, where there was agreement and disagreement within the same group (e.g. health providers cite different drivers of informal payments), and where there was agreement and disagreement across types of interlocutors (e.g. health providers stated informal payments had stopped and MSAM women disagreed).

MS then discussed conclusions with JD, triangulated the emerging findings among different theories and past empirical findings, and triangulated among methods, assessing to what extent the participant observation, project reports, and FGDs suggested similar conclusions [45].

## Results

### Project implementation

A description of the pilot phase of this project has been presented elsewhere [14], and other papers discuss the information and communications technology (ICT) component of the project in depth [50, 51]. In this paper, we thus focus on the ways in which SAHAYOG and partner CBOs adapted the project strategy over time in order to set the stage for deeper analysis on program outcomes and context.

SAHAYOG and the CBOs made program changes based on feedback from MSAM and the CBOs. These changes were intended to ensure that women reported demands for informal payments, that providers saw and reacted to the data, to shift the incentives facing providers and managers, and to respond to the changing political context. The changes made included making the hotline more functional and accessible, increased campaigning to generate more complaints to the hotline, reporting back to the community on the number and type of complaints, greater engagement with hospital committees, proactively providing the data displayed on the MS, MA website to health sector stakeholders, more deliberate media outreach, and institutionalizing health facility dialogues.

Despite these changes, SAHAYOG and the CBOs discontinued the hotline in December 2015. They made this decision after conducting an analysis of the relatively high operational costs, the low number of complaints generated, and the seemingly less than desired impact on the incidence of demands for informal payments. They decided that further investment was not efficient, particularly given the fact that the state government had started their own hotline where community members could report having been asked to make an informal payment. The government explained that this hotline was inspired in part by MS, MA. Since SAHAYOG and the CBOs had conclusively established the problem of informal payments, they decided that further data collection would not necessarily be helpful. They continued to try to influence state and local level policy and practice by discussing the data at various official fora in which they participated.

### Project impact

In keeping with SAHAYOG’s, the CBOs’, and MSAM’s respective missions and our research questions, we group our findings and discussion into three broad categories: (1) impact on empowerment, (2) impact on the incidence of informal payments and related issues, and (3) macro- and micro-contextual determinants of health system responsiveness.

1. Impact on empowerment

We use Kabeer’s [52] concept of empowerment, “the process by which those who have been denied the ability to make strategic life choices acquire such an ability” (p. 1). Making choices entails exercising three inter-related capacities: resources (preconditions), agency (process), and achievement (outcomes) [52]. For the purposes of this study, we focus primarily on the knowledge components of resources, secondarily on the relationships component of resources, and on the negotiation, voice, and mobility elements of agency. Achievements refers to the accomplishment of desired goals, namely the cessation of demands for informal payments and other improvements in the inter-personal and clinical quality of care. We address resources and agency here, and describe achievements in the section describing MS, MA’s impact on the incidence of demands for informal payments.

As documented in earlier studies, over the course of their approximately ten year engagement with MSAM, MSAM women, particularly those who had been members for a long time, have gone through repeated consciousness raising processes, as they learned about their rights and entitlements and successfully addressed priorities in multiple domains, such as distribution of subsidized rations and the minimum rural employment guarantee. As low caste, poor women, new members often initially did not think of themselves as rights holders, or as having the “right to have rights” [41, 53]. This self-perception evolved over time as MSAM women came to believe that health and social outcomes are not due to chance, but are matters of social justice which the state has the responsibility to address [41].

In interviews and FGDs comprising the current study, SAHAYOG and CBO staff and MSAM women themselves emphatically communicated the multiple ways that their resources and agency had increased. They described a general sense of empowerment and loss of fear from their involvement in MSAM, as well as gains in knowledge and confidence specific to the health sector. Sometimes, they discussed MS, MA specifically, but more often, they referred to their engagement in MSAM in more general terms (i.e. not limiting themselves to the life of the MS, MA project).

*We have abandoned our fear from the day of joining the Forum [MSAM]. We were afraid of speaking out in the past. Now, we can talk to the Chief Medical Officer and speak from the stage using a microphone… our fear is finished now. (FGD with MSAM women)*

Women generally attributed their increased agency to the knowledge resources they had gained through MSAM.

*When they demand money, we say that it is against the rules. Then, they realize that we are the members of MSAM. Those who are not members of MSAM cannot speak. They do not have information. (FGD with MSAM women)*

Many women exhibited agency in their willingness to put themselves in uncomfortable, adversarial, or risky situations to assert their knowledge and claim rights. Several CBO staff and MSAM members described situations where women faced down threats in their refusals to make informal payments. MSAM women reported a variety of menacing situations, including providers threatening physical violence, mobilizing political allies against the family making a complaint, filing a false legal complaint against the woman concerned, being rough with the laboring woman and/or the newborn, and, denying the woman care.

*Since they have to take money from us, they behave properly. They misbehave only if you refuse to give them informal fees. The enmity starts when we refuse to bribe them. They refuse to prepare a record of treatment if we do not pay informal fees… People do have a fear that if they refuse to make informal payments, doctors may kill our patients by poisoning them. (FGD with MSAM women)*

Our data did not reveal any allegations of actual poisoning (just the fear of it), but we did hear multiple stories of women who identified as MSAM members being denied care, suggesting that providers punished those who tried to claim their rights. Sometimes women were able to negotiate to receive care anyway, sometimes not. For example, an MSAM leader reported accompanying her laboring daughter-in-law and being recognized as an MSAM leader by the Medical Officer in Charge. He refused to provide care, and the MSAM leader countered that she was going to call his boss, the Chief Medical Officer; the provider relented.

Many other MSAM women referred to this relational resource of access to frontline providers’ “bosses,” whom they had met at dialogue events or contacted in the process of making complaints.

SAHAYOG and CBO staff emphasized that this improved knowledge resources and agency resulted from a long-term process, and was not just the result of the MS, MA project. MSAM members, too, understood changes in their resources and agency in the context of their longer-term engagement with MSAM.

*It has been ten years since we are associated with the organization. It was essential to join it…. We learned how to register our complaint in Lucknow through mobile phones. We also learned about human rights. Initially, we were fearful but now we can threaten the ASHA [community health worker] and ANM [frontline midwife who supervises the ASHA] in the name of registering a complaint. (FGD with MSAM women)*

Empowerment does not occur solely in relation to the health system; MSAM women claimed their rights in a dynamic context of gender, caste, religious, and political relations. There is widespread agreement in the empirical literature on social accountability and participation that women’s political capabilities are mediated by gendered social norms within the household and the community [32, 54, 55]. Although most of the discussions in our interviews and focus groups centered on interactions with the health system, MSAM women regularly referred to these wider social norms. They indicated that the empowerment they felt was not just vis-à-vis the health system. Some – though not all - noted that they felt liberated from husbands and/or mothers-in-law who were opposed to their mobility and to their engagement in political matters outside the home.

*Family members discourage us. When I came to the meeting for the first time, I had informed my husband. But, when I went back, he slapped me. When I argued, he started beating me, and kicked me out of the home. Then some of the members went to my home and convinced him. (FGD with MSAM women)*

In sum, in our research sites, MSAM women manifested increased resources and agency; they developed this over a time frame well before MS, MA began, and they sometimes fought to claim their rights despite significant opposition.

(2) Impact on the incidence of informal payments and related issues

Kabeer describes resources, agency, and achievement as indivisible components of empowerment [52]. SAHAYOG and the CBOs explained that they felt that increased knowledge and opportunities to use that knowledge were important goals, but they also realized that failure to impact the incidence of demands for informal payments (achievement) would undercut community engagement, and ultimately empowerment. In FGDs, MSAM women agreed among themselves that significantly reducing the frequency of demands for informal payments was a key objective.

MSAM women described being asked to make informal payments to receive maternal care, to see the newborn, to be discharged from the facility, to take the ambulance to the facility to deliver, to receive the JSY check (a conditional cash transfer for giving birth in a facility), and for the labor room to be cleaned. As summarized in Table 1, a review of all cases reported over the life of the project indicated that a total of 2850 reports were made to the hotline for several reasons.

**Table 1:** Summary of MS, MA cases from January 2012–December 2016

Category of complaints	Percentage of reports (%)
Bribe for admitting the patient, treatment or during delivery	23
Money asked for medicine, gloves, soap	21
Money asked for ambulance service	20
Money asked in order for the patient to receive the JSY cheque	15
Bribe for examination	14
Money asked for blood or operation	7

Almost 72% of all payments reported to the hotline were for more than 500 rupees. Many were asked to procure from outside pharmacies medicines that the health facility was mandated to provide. SAHAYOG and the CBOs believe that in many of these cases, private pharmacies provide kickbacks to the prescribing providers – a phenomenon that has been widely reported in India [56] though in some cases the health facility may actually be stocked out of the drug in question.

SAHAYOG, the CBOs, and MSAM women successfully educated or convinced some state, district, and facility staff about the frequency and impact of informal fees. As a result of what they learned and/or the pressure SAHAYOG and the CBOs brought, our data indicated that these officials made administrative allowances to address informal payments, such as issuing orders mandating discussion of MS, MA data in regular meetings, and mandating CBO participation in various forums. For example, the Uttar Pradesh NRHM Mission Director issued a letter asking that Patient Welfare Committees discuss MS, MA data at their meetings. Chief Medical Officers reissued this letter to Medical Officers in Charge of health facilities, asking them to ensure implementation at facility level. One District Program Manager reported that the facilities in his jurisdiction outsourced diagnostic tests to prevent demands for informal payments for laboratory tests.

According to CBO and MSAM women, district and block level dialogues resulted in short-term (2 week – 3 month) reductions in demands for informal payments from MSAM and non-MSAM patients, as well as improvements in other domains that had been raised at the dialogue. When asked what factors precipitated these improvements, the CBO and MSAM women widely agreed that these changes were more likely to occur when many women attended the dialogue, as well as when higher-level officials attended. Improvements went beyond informal payments to also include better (more timely and/or free of charge) ambulance service in rural areas; the installation of solar lights and generators in remote facilities; cleaner facilities; new equipment in maternity wards; and the provision of free food to in-patients, as stipulated by policy. Multiple interviewees from different stakeholder groups recounted that some health system managers asked frontline health providers to explain why MS, MA data showed persistent demands for informal payments. In some cases, hospital staff reportedly returned money to patients. Due in part to the public nature of the district or block dialogues, the CBOs explained that they could shame providers into participating in the dialogues and to following through on commitments made during the dialogues.

The hotline also included an emergency number for urgent cases; this emergency line resulted in immediate aid for callers. The emergency number was staffed 24 h per day by CBO employees. The interviews and FGDs revealed many examples of emergencies being addressed, often because the CBO representative on call then contacted someone above the offending provider in the hierarchy. For example, a woman being denied a blood transfusion, a skeptical woman being told she needed an urgent cesarean section and she needed to pay for it, and multiple women being denied care because they were allegedly presenting at the health facility “too late” in their deliveries, had their problem addressed immediately after contacting the emergency line. SAHAYOG and CBO staff reported that this immediate responsiveness helped to maintain community support for the project.

SAHAYOG staff explained that because the project entailed regular CBO and SAHAYOG interactions with health sector officials at block, district, and state level, the CBOs and SAHAYOG enjoyed greater visibility and cooperation vis-à-vis official structures. SAHAYOG input was regularly solicited by state level health authorities. SAHAYOG employees reported that they felt that most policy-makers perceived SAHAYOG as an organization providing relevant, authentic data from the grassroots.

Against this backdrop of increased knowledge and commitment at the mid and upper levels of the state health system, public dialogues, and increased SAHAYOG and CBO engagement in policy-making and policy monitoring, interviewees of all types reported that MSAM women who asserted their rights were mostly able to avoid making payments, though some faced retaliation.

*When I took my daughter-in-law to the hospital… the staff demanded 500 rupees and I was asked to go and buy a medicine from outside. When I scolded them, the Auxiliary Nurse Midwife [ANM] started arguing and said that things may go out of stock anywhere. Then I replied that you should take care of the things going out of stock and should bring them before they are finished. I also made a telephone call to the CBO… [the ANM] refused to talk [to the CBO] but she also abandoned her demand for money (FGD with MSAM women)*

MSAM women and CBO representatives explained that non-MSAM women accompanied by MSAM women (or by a representative of the local CBO) were also largely able to avoid payments. These changes spilled over to the general population in limited contexts; for example, FGD participants reported that lower caste women – MSAM members or not - were now less likely to be asked to pay for the cleaning of the labor room after delivery, a practice that had been routine. As testament to MSAM’s informal regulatory power, in some facilities, health facility staff tried to obtain explicit MSAM member support for urgent patient referrals, in order to show that the transfers were needed and consensual.

However, despite the reported facility-level changes following dialogues, SAHAYOG and the CBOs’ increased participation in policy discussions, and MSAM member ability to refuse to make informal payments, MSAM, CBO, and SAHAYOG representatives agreed that they did not accomplish their ultimate goal of reducing demands for informal payments on a population level; there was little system change. The CBOs and MSAM reached this conclusion by informally asking women about payments after they left health facilities, and by discussing what they heard from friends and neighbors at MSAM meetings. In an FGD, an MSAM member described this lack of progress:

*Many of us went to the hospital, we demanded that informal money should not be taken from us. We had several meetings on these issues. Staff say that they will not take money again but things go back on the same track once again. Doctors have assured us many times that they will punish those who demand money. But, everyone is a culprit there. (FGD with MSAM women)*

Participants summarized the situation similarly at an FGD for CBO members. Those who know their rights can sometimes avoid paying, but others cannot:

*Interviewer: Is there any change in the situation? Do such incidents occur less now?*

*Respondent 1: Staff cannot take money from those who know their rights and entitlements. Such people fight and do not pay informal money. Otherwise, they demand [money] in the name of celebration...*

*Respondent 2: Those who fight until the end can save their money, but not all of us are able to do that. (FGD with CBO members*)

Reporting to the hotline went down over time; SAHAYOG and CBO staff explained that this was likely because the campaign seemingly had little impact on the likelihood of women being asked to make informal payments. The campaign was based on the premise that the data could foment system level change. Not seeing improvement, women were less motivated to report, and were less optimistic about their ability to effect change. Many expressed the following sentiment in interviews and discussions:

*We are now tired of attending dialogues and complaining. Everything becomes the same after a temporary change. (FGD with MSAM women)*

As SAHAYOG and CBO staff explained in interviews, decreases in reporting gave the government an excuse to claim that demands for informal payments had decreased. The hotline began to slide into irrelevance, as fewer women reported or were motivated to do the risky work of complaining about informal payments.

(3) Macro and micro contextual factors shaping state responsiveness

Despite their increased knowledge and relational resources, agency, and past achievements in many domains, such as in securing better access to rations for the poor, ensuring the regularity of Village Health and Nutrition Days, integrating MSAM members into Village Health and Sanitation Committees, and securing greater access to the minimum employment guarantee, MSAM women had fewer achievements in the domain of informal payments [57]. Health providers and the system overall were only partly responsive to demands regarding informal payments.

The contextual factors shaping state responsiveness are less explored than the community dynamics of social accountability, though response is germane to the success of an effort and to continued engagement from the community [17, 31, 58]. In this section, we discuss macro and micro features of the context that seemingly influenced health system responsiveness related to informal payments. The contextual factors that arose from our data are broadly similar to what has been identified as contextually relevant in other settings, including “broad features of the political economy” [28, 59]. Our data revealed relevant factors that are manifest at national and subnational levels, including caste hierarchies, provider commitment to ending informal payments, the embeddedness of informal payments in the health system, human resource scarcity, the overlapping private interests of pharmaceuticals and providers, differences in regional development, and individual influence on the project.

#### Caste

Many MSAM, CBO, and SAHAYOG interviewees indicated that lower caste women had fewer opportunities to exercise agency and to realize achievements than higher caste women, as decision-makers did not give equal consideration to the rights and opinions of lower caste women. As explained by a Dalit MSAM woman:

*If N [a higher caste woman] complains about something, people are not going to say anything. But, if I make the same complaint they will call me all sorts of names and hurl abuses at me. (FGD with MSAM women)*

None of our interviewees or FGD participants suggested that caste make up per se explained differences between project sites. However, CBO and SAHAYOG staff explained in interviews that lower caste and scheduled tribe women were at a disadvantage in all arenas of rights claiming, and were generally more likely to be asked to make informal payments, because health providers perceive that lower caste women have less resources and agency. Moreover, the participants in one FGD indicated that heterogeneous MSAM groups may have been less durable. Some lower caste members of one MSAM group explained that they no longer met without explicit CBO facilitation and support. They stated that the elected head of this group was lower caste, and upper caste women would not attend meetings she called without the external coordination and legitimacy conferred by CBO engagement.

#### Provider commitment to ending informal payments

As noted, there was some evident high-level commitment to decreasing informal payments at the state level, as NHM leadership expressed their support for the project and asked for SAHAYOG input into multiple policy-making processes. This commitment was buttressed by changes in the political context, as, shortly after the project began, newly installed policy-makers relaxed restrictions on NGOs and focused more resources on fighting corruption. When district officials were asked why this central level commitment did not engender commitment among frontline providers, they referred to technocratic and logistical barriers, such as lack of required computer skills to look at the MS, MA website and lack of time. District officials, SAHAYOG, and the CBOs also cited poor communication regarding policy priorities between the central level of the state and districts. However, our research also suggested that some frontline providers and medical officials seemingly did not change their behavior because they did not agree that informal payments were deleterious. About half of the district officials and senior managers interviewed disagreed with the premise that informal payments were problematic, stating that it was acceptable for service providers to demand informal payments, as poor women received a conditional cash transfer if they delivered their baby in a health facility, and, if women were not asked to pay, they would overuse medical care. Several claimed that women’s expectations were too high, or, that if women did not want to make informal payments, they should simply refuse to pay them.

#### Embeddedness of informal payments in the health system

Interviewees and focus group discussion participants offered other reasons for the persistence of informal payments, with many interlocutors of all types converging on one key point: there is a complex nexus of financial exchanges that few providers and managers are motivated or able to change. Informal payments are deeply embedded in the health system, such that it may be more difficult to obtain responsiveness in this domain than in the other areas SAHAYOG, the CBOs, and MSAM had worked.

Among providers, several phenomena feed the nexus. First, as has been detailed in the peer-reviewed literature, interviewees of various types explained that many providers pay for their position [60–62]. Providers thus wanted income from informal payments in order to pay to stay where they are, or to pay for a more desirable posting. Second, interviewees from all stakeholder groups speculated that providers who levied informal fees were financially indebted to their superiors or to other decision-makers, perhaps for obtaining a post or for superiors’ overlooking transgressions. Third, the nexus could expand, neutralizing potential whistle blowers or opponents. For example, SAHAYOG and the CBOs explained that they initially assumed that Accredited Social Health Activists [ASHAs] would be natural allies of MSAM and MS, MA. However, ASHAs rarely showed themselves to be allies, and some SAHAYOG and CBO staff concluded that the principals of the nexus co-opted ASHAs by allowing them to charge their own informal fees. We did hear scattered reports from SAHAYOG, CBOs and MSAM women of ASHAs preventing CBOs from entering villages to conduct awareness raising on entitlements and the MS, MA project. ASHAs may have been more vulnerable to co-optation because the amount of compensation they received from the government had decreased in recent years; several CBO and MSAM respondents reported that they thus became more willing to seek payments directly from women.

Mid and senior level managers claimed that because of the nexus, their ability to sanction the providers demanding payments was limited. The nexus was not confined to a health facility; financial relationships often related to broader political dynamics in the community. Thus, political power could be deployed to maintain the status quo. Indeed, political patronage was ubiquitous in discussion, with multiple managers claiming that they were unable to sanction employees who were below them in the hierarchy because these individuals had political connections they could use to get the manager transferred. Providers who had lived in a region longer reportedly had stronger political connections, making them more impervious to discipline. The nexus seemingly had the effect of flattening the hierarchy for most (not necessarily for those with the least power, such as ASHAs) such that everyone owed everyone something; as one district official in Azamgarh described, “everyone has a jack” he can deploy to avoid accountability. Some managers reported disciplinary workarounds, such as “managing on the inside,” or resolving the problem by reaching a compromise with the employees concerned – sometimes by dividing up the spoils.

MSAM women, in contrast, had few political connections, and few “jacks” to deploy.

*Interviewer: Is it more important to have a good connection with some influential people than complaining?*

*Respondent: Yes. Nothing is possible when you do not have a strong connection. (FGD with MSAM women)*

#### Human resource scarcity

Officials and managers stated that the nexus was even stronger in a setting of very limited human resources. For example, one district official explained that he was not in a position to punish a doctor who violated policies in the understaffed hospital. Providers in such contexts are in a strong negotiating position. A District Program Manager from Azamgarh elaborated more fully:

*We cannot take direct action against the staff, as the number of staff is already less than what we require. If we did [take action], the services which are available today would not be available tomorrow. They have built up a strong nexus among themselves. We cannot take any action against any of them; if they are suspended for even two three days then we won’t be able to provide even basic facilities to our clients. We could have taken action only if there were a good number of doctors available there. (IDI with District Program Manager)*

While there was widespread agreement among different types of interviewees and FGD participants that a nexus existed and that this nexus nurtured informal payments, we interpret manager explanations of why they were unable to address informal payments with some skepticism. SAHAYOG and CBO staff speculated that managers try to attribute informal payments solely to lower level staff in order obscure their own role in it. Some managers may benefit from informal payments indirectly or directly, or they may engage in their own corruption that health providers know about, so they do not dare to stop frontline providers. A limited number of managers substantiated this view, as they explained that demands for informal payments at the point of service was a visible form of corruption that patients saw, but that it was the last link a long chain of corruption. Informal discussions during participant observation confirmed this view. Providers noted that there was corrupt behavior throughout the system; demands for informal payments were among the more visible manifestations of corruption.

#### Overlapping private interests of pharmaceuticals and providers

The nexus also applied to the prescription of medicines from outside pharmacies, which was one of the most ubiquitous forms of informal payments. To decrease patient opposition to purchasing these drugs, it appears that providers fed widespread myths that government-supplied generic medicines are of poorer quality and less effective. MSAM members and families of patients encountered during the participant observation reported that they were willing to purchase ‘quality’ medicine from the outside. While we did not explore this angle in our research, recent research in India suggests that pharmaceutical companies also propagate this false narrative, while independent testing reveals that government supplied generics are of comparable quality to branded medications [63].

#### Differences in regional development and individual influence on the project

The findings regarding regional differences and individual influence are intuitive, so we do not describe them in-depth here. The key point is that there were regional differences in project uptake and buy-in, with the more geographically remote district seeing less impact. Finally, some successes in reducing demands for informal payments were partly attributable to individual people, both within the CBO and the Indian Administrative Service, the professionalized bureaucracy of India.

## Discussion

In the context of increasing focus in global health and development on how civil society and advocates can create and leverage countervailing power to make health systems more accountable, the MS, MA experience offers important lessons and questions.

SAHAYOG and the CBOs nurtured MSAM member relational resources and agency in part through public dialogues. Public dialogues were structured according to rules that tried to minimize the relevance of embedded hierarchies; women of all castes and classes were theoretically able to speak to providers and district officials. These facilitated interactions with health providers and policy-makers constituted a new ‘social space,’ wherein marginalized women could enact new identities nurtured by MSAM [35, 64]. Over time, repeated interactions such as these can “socialize the poor into potentially constructive relationships with…the policymaking state” [54, 65]. This increased access to the state apparatus served as a key resource, increasing MSAM women’s negotiating leverage. They described utilizing this resource in public dialogues, where they interacted with “bosses” and provided personal testimony regarding demands to make informal payments and other negative experiences with the health system.

However, despite increased resources and agency, informal payments proved to be resilient. They are “vertically integrated;” the phenomenon of informal payments is shaped at multiple levels of the health system [66]. Many public sector problems are political and vertically integrated, but our data regarding informal payments and MSAM’s more successful efforts to improve public service delivery in other domains suggest that informal payments are especially embedded. In a context where many providers may disagree that the stated target of the campaign – informal payments – are a problem and where they are embedded in everyday practice, most people working in the system are unwilling to use their “jack” to address this particular issue. Thus, the nexus presents a collective action problem: health workers face pressure to continue demanding informal payments, as these payments are a central part of the way the health system functions [67]. Providers were dis-incentivized to stop demanding informal payments, as this could anger colleagues who expect spoils to be shared, and put them at a material disadvantage if they wanted to seek better postings. Women, too, may have reason to continue to make informal payments, as those who do pay may receive better quality care more quickly. This segmentation of the patient market between those who can and those who cannot make informal payments has been documented in the empirical literature globally, with those making payments often feeling they have little choice but to pay [13].

As a vertically integrated phenomenon, informal payments are closely related to the dynamics of economic and political power in Uttar Pradesh. We do not mean to suggest that health system employees have no professional or moral values or that formal rules are of no consequence. Rather, we learned how informal norms, or what Jean Pierre Olivier de Sardan describes as “practical norms” are also quite salient. Practical norms are not explicit in public discourse or acknowledged in “official moral rhetorics” [68], yet they are “relatively convergent and recurring,” or, as we have described, embedded in everyday health system practice [69]. Social accountability efforts thus can be understood to occur “not in the absence of the ‘standard model’ of bureaucratic and political accountability, but among its ruins and/or in the gaps it leaves” [70].

Our findings regarding the particularity of informal payments is apiece with empirical literature concluding that the nature of the challenge to be addressed by social accountability efforts shape health system responsiveness and willingness to engage in the project [17, 71, 72]. A recent synthesis of health provider responsiveness to social accountability efforts in health in low- and middle-income countries found that corruption and quality of care for poor women was a common domain of failed responsiveness [17]. Among other attributes, failure was more likely in contexts where providers: view patients as users, rather than citizens; do not fear repercussions from important third parties; and do not feel morally obligated to address the issue at hand [17]. These are all general trends across countries and contexts, but it is noteworthy that they were largely corroborated in our research.

Our study proffers new questions and propositions for further research. First, MS, MA was embedded in a long-term effort to increase poor, low caste, Muslim, and Tribal women’s empowerment. Almost all interviewees reported that resources and agency had improved over time, and that there had been greater achievements in domains other than informal payments. Thus, rather than thinking of MS, MA as a failed project, we can think of it as a less successful component of a much larger effort. Second, SAHAYOG employed a vertically integrated approach, and they were arguably fairly successful in shaping policy and processes at state level. Yet, some SAHAYOG staff mentioned that their increasing success in state government engendered resentment at the facility level. How to generate countervailing power for such issues – particularly when they affect a politically marginalized segment of the population – is not apparent. It is possible that more of the same – more media coverage, more dialogues to share experiences with providers and to enact new identities, and more pressure on decision-makers – would generate more change. It is also possible that particular strategies for embedded collective action challenges such as informal payments are needed. This might include specific efforts to support labor organizing and alliances with frontline providers, campaigns to fund the health sector adequately, and, in the case of electoral democracies such as India, broad-based electoral campaigns.

Third, social accountability has been criticized for possible elite capture [28]. There is little literature in social accountability about the distribution of the benefits to various groups, but much of the literature describes general community processes that may exclude just the type of women who join MSAM. Community level processes are often dominated by village leaders and others with more power, with those filling gender and other quotas often accorded little more than a token role [70]. MS, MA avoided this problem, in part by explicitly targeting the most marginalized, and by focusing on violations (i.e., being asked to make an informal payment) rather than a list of priorities identified through community deliberation, which is much more vulnerable to capture. This focus on the non-elite may explain some of the particular challenges MS, MA faced and suggests that the project may be somewhat distinct from much of the growing evidence base on social accountability for health. Fostering increased resources and agency was a long process, with many women initially not perceiving themselves as rights holders. Changes were manifest in the social space of public dialogues, and powerfully in patient assertions of rights in interactions with providers. Yet, providers have not necessarily embraced this notion of low caste, tribal, or Muslim women as citizens with entitlements, especially in the context of free government services for childbirth.

Our study has several limitations. First, it was conducted post hoc. This allowed us to learn about the evolution of the project and to assess impact after project activities had been completed. However, due to the limited nature of project record keeping, we were unable to try to make links between rates of reporting and specific projects events, such as dialogues. Second, our central focus was MS, MA, not informal payments. Over time, it became clear that the dynamics of informal payments merited further investigation; we did not undertake an in-depth review of this phenomenon in India. Third, some of our minor conclusions are tepid, as there was disagreement among the interviewees, or because only one or two interviewees raised a given point. For this reason, we clearly state in the manuscript how many and which type of interlocutor raised a particular point. We included some tepid conclusions here because they were novel in the literature and merit further exploration.

## Conclusion

SAHAYOG and two CBOs launched MS, MA to address an intransigent problem that MSAM women had identified as a priority. Over the life of the project, SAHAYOG and the CBOs adapted their strategy to enhance project impact. MS, MA engendered greater resources and agency for MSAM women; women learned about their entitlements, had access to mechanisms for complaints, and, despite risk of retaliation, many MSAM women felt capable of demanding their rights in a variety of fora. These women did achieve successes in that MSAM members were mostly able to avoid making informal payments to the health sector, but they largely were unable to affect this change for other women in the community at large. MSAM women and the CBOs perceived their work on informal payments to be somewhat unsuccessful.

In brief, SAHAYOG assumed a supremely difficult task. Project strategy changes may have made the task somewhat less difficult, but given the population making the rights claims and the rights they were claiming, a strategic, watchdog approach may require a much larger and stronger coalition, thus generating more than rhetorical commitment and scattered genuine commitment among health system actors.

## Data Availability

The datasets generated and analyzed during the current research are not publicly available as individual privacy could be compromised, but they are available from the corresponding author on reasonable request.

## Abbreviations

ANM: Auxiliary nurse midwife
ASHA: Accredited Social Health Activist (type of community health worker)
CBO: Community-based organization
FGD: Focus group discussion
ICT: Information and communications technology
IDI: In-depth interviews
IRB: Institutional Review Board
IVR: Interactive voice response
JSSK: Janani Shishu Suraksha Karyakaram
KII: Key informant interviews
MS, MA: Meri Swasthya, Meri Awaz (My Health, My Voice in English)
MSAM: Mahila Swasthya Adhikar Manch (Women’s Health Rights Forum in English)
NGO: Non-governmental organization
NHM: National Health Mission
NRHM: National Rural Health Mission
